# Design of a Monolithic Double-Slider Based Compliant Gripper with Large Displacement and Anti-Buckling Ability

**DOI:** 10.3390/mi10100665

**Published:** 2019-09-30

**Authors:** Guangbo Hao, Jiaxiang Zhu

**Affiliations:** School of Engineering-Electrical and Electronic Engineering, University College Cork, T12 K8AF Cork, Ireland

**Keywords:** compliant gripper, large motion, anti-buckling, modelling

## Abstract

In a micro-manipulation system, the compliant gripper is used for gripping, handling and assembling of objects. Large displacement and anti-buckling characteristics are desired in the design of the gripper. In this paper, a compliant gripper with these two characteristics is proposed, modelled and verified. The large displacement is enabled by using distributed compliance in a double-slider kinematic mechanism. An inverted flexure arrangement enables the anti-buckling of the gripper when closing the two jaws. A pseudo-rigid-body model (PRBM) method with the help of virtual work principle is employed to obtain several desired analytical relations including the amplification coefficient and kinetostatics. The results of the finite element analysis (FEA) are shown to be consistent with the results of the derived analytical model. An experimental test was carried out through a milling machined aluminium alloy prototype, the results of which verify the good performance of the compliant gripper.

## 1. Introduction

Compliant mechanisms use the elastic deformation of their compliant/flexible components to complete the transformation of motion and force. Compliant mechanisms are widely used in minimally invasive surgery, micro-switch technology in communication, precision positioning motion, precision and ultra-precision manufacturing, and micro-electromechanical systems (MEMS) [1,2], due to their well-known merits in high performance and low cost [3].

At present, pseudo-rigid-body models (PRBMs), finite element methods and topology optimization are commonly used for designing compliant mechanisms. Howell and Midha first introduced the concept of PRBM [3]. The PRBM method means that the emerging rigid linkage mechanism analysis method can be used to study the kinematics of the corresponding compliant mechanisms [3]. The principle of topology optimization is to divide the structure into several sub-regions, and to analyse the structure of these sub-regions. Then some sections are deleted according to specific optimization criteria [4,5].

The design of compliant grippers for micro-manipulation has been carried out to obtain promising results in balanced-force gripping [6,7,8], nanonewton force-controlled manipulation [9], autonomous robotic pick-and-place [10], amplifying mechanisms and novel actuation methods [11,12,13]. A typical compliant gripper consists of an input port actuated by a micro-motion actuator, and two jaws (as output ports) with parallel synchronised opposite motions to close [14,15,16,17,18]. In order to deal with a wide range of dimensions of objects, the motion displacement (output) of jaws is expected to be large [14]. The large output displacement requires a gripper design with this displacement capability, as well as an input actuator that can enable this output [19]. This paper concentrates on the gripper body design, rather than actuator selection and system integration. 

Without compromising the compact footprint of a design, the use of distributed compliance of enables the large displacement, based on the stress criteria, while not creating a large actuation force (i.e., energy-friendly design). In addtion, the distributed compliance does not introduce much stress concentration that alleviates the fatigue issue. Nevertheless, the introduced distributed compliance is prone to result in buckling when the compliant members suffer from a large compression force. The buckling can limit the motion range of the jaws without breaking the stress limit. Furthermore, the buckling can also make the gripper incapable of imposing large force (if required) on the target object, which limits its applications in high-payload handling and crushing oriented purpose. There are few reports that have addressed this buckling issue for the gripper design.

Motivated by the above issues in compliant gripper design, this paper aims to design a monolithic compliant gripper with a large displacement and without buckling in the closing operation. This paper is organised as follows. The compliant gripper with the desired performances is presented, followed by the derivation of the analytical model in Section 2. Section 3 investigates the finite element analysis (FEA) and experimental tests. Comparisons of results are provided in Section 4. Discussions and conclusions are finally presented in Section 5 and Section 6, respectively.

## 2. Mechanism Design and Modelling

### 2.1. Design of Compliant Gripper

**Figure 1:**
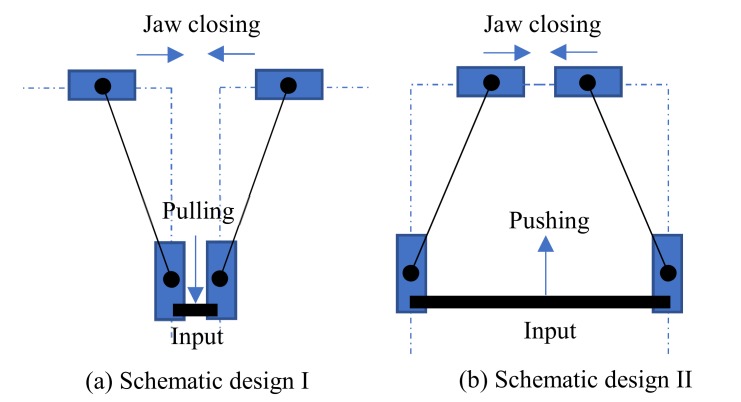
Two schematic design using double-slider compliant mechanism.

There are some kinematic mechanisms that can be used to conceptually design the compliant grippers, such as the double-slider mechanism [19] and the straight-line mechanism [15]. Here, the double-slider mechanism refers to the PRRP mechanism where P denotes a prismatic joint and R denotes a revolute joint. The double-slider mechanism is probably the easiest/simplest one to deploy a compliant gripper [19]. Two schematic designs of compliant grippers, which use two mirror-symmetrical double-slider mechanisms with connection at the input, are shown in Figure 1. One difference between the two designs is the input/actuation direction for closing jaws, where Figure 1a requires a pulling force (input) for closing jaws (output) while Figure 1b requires a pushing force for closing jaws.

**Figure 2:**
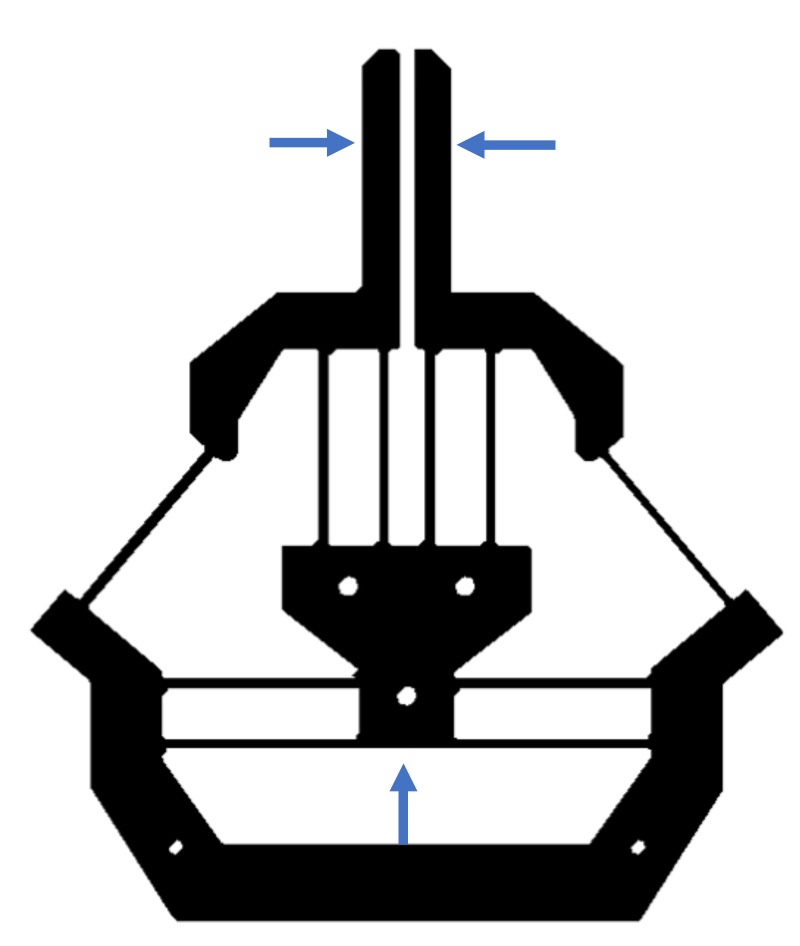
The first version of compliant gripper.

Considering a design that has a universal suitableness for various actuators, the input force is desired to be a pushing force for closing jaws. Most popular actuators (such as the pizeoelectric/lead zirconate titanate (PZT) actuator [20,21] and the pneumatic actuator [22]) being used in compliant mechanisms are often efficient for pushing, yet less efficient for pulling. For example, a PZT actuator is fragile in tensile stress. Therefore, Figure 1b is a better candicate to guide the design of compliant grippers from the pushing actuation point of view. Replacing each P joint with a compliant parallelogram mechanism, and the RR chain with a fixed-guided compliant beam, we can obtain the first-version of the compliant gripper, as shown in Figure 2a.

**Figure 3:**
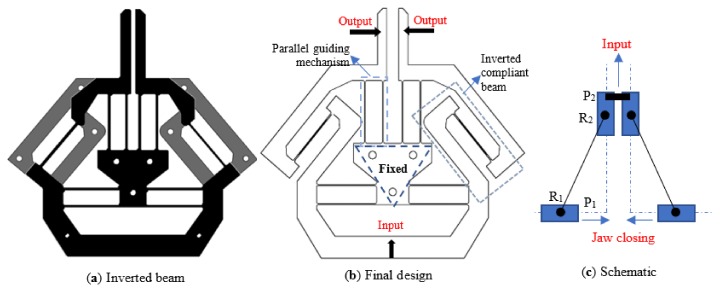
The final design of the compliant gripper.

As mentioned in the introduction, the buckling should be avoided in the gripper design when the input force is a pushing force. However, in the design shown in Figure 2, the pushing input force will lead to compression force on the fixed-guided compliant beams, which can not address the buckling issue. The solution presented in this paper is to invert the fixed-guided beam (Figure 3a) to introduce tension forces only on the fixed-guided compliant beam when the input is a pushing force (Figure 3b). The kinematic principle of the final gripper is shown in Figure 3c, which will be detailed in the next section.

### 2.2. Kinematic Analysis

**Figure 4:**
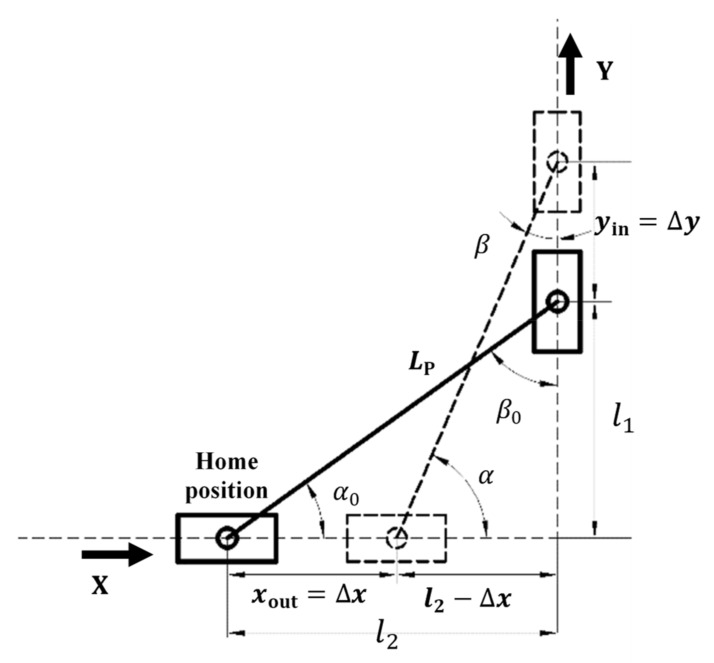
Kinematic representation of the double-slider mechanism.

Under the assumption that the elastic deformations in the gripper only occur in the compliant members and that the rest are rigid, and based on the PRBM principle, the left half of the compliant gripper (Figure 3b) can be kinematically represented as shown in Figure 4.

We can first obtain the motion displacements of each joint with regard to its home position (no flexure deformation) as below:

(1) $$\Delta \alpha =\arcsin\left(\frac{l_{1}+y_{\mathrm{in}}}{L_{P}}\right)-\alpha_{0}$$

(2) $$\Delta \beta =\beta_{0}-\arccos\left(\frac{l_{1}+y_{\mathrm{in}}}{L_{P}}\right)$$

(3) $$y_{\mathrm{in}}=\Delta y\;$$

(4) $$x_{\mathrm{out}}=\Delta x=l_{2}-\sqrt{L_{P}{}^{2}-{\left(l_{1}+y_{\mathrm{in}}\right)}^{2}}\;$$

where Δα and Δβ represent the angle changes/displacements of $R_{1}$ and $R_{2}$ joints, respectively. *y*_in_ is the displacement of P_2_ joint as the input, and *o*_ut_ is the displacement of P_1_ joint as the output. $\alpha +\beta =\alpha_{0}+\beta_{0}=\pi /2$ and $l_{1}{}^{2}+l_{2}{}^{2}={\left(l_{2}-\Delta x\right)}^{2}+{\left(l_{1}+\Delta y\right)}^{2}=\;L_{P}{}^{2}.$ All the symbols are labelled in Figure 4.

The ratio between the output displacement and the input displacement can be obtained:

(5) $$\frac{x_{\mathrm{out}}}{y_{\mathrm{in}}}=\frac{l_{2}-\sqrt{L_{P}{}^{2}-{\left(l_{1}+y_{\mathrm{in}}\right)}^{2}}}{y_{\mathrm{in}}}\;\;\;\;$$

It can be seen from Equation (5) that the amplification ratio is not constant. Under the assumption of a small displacement, the amplification ratio equation can be expressed as:

(6) $$\frac{x_{\mathrm{out}}}{y_{\mathrm{in}}}≅\frac{l_{1}}{l_{2}}=\tan\alpha_{0\;\;}$$

From Equation (6), it can be learned that the double-slider mechanism has an amplification factor that is dominantly controlled by the angle *α*_0_. In this paper, an amplification ratio between 1 to 2 is selected so that the input displacement and output displacement are equally easy to measure.

### 2.3. Stiffness Analysis

Stiffness analysis was carried out to obtain the relationship between the force and the displacement of each compliant joint.

The linear (primary) stiffness of the compliant parallelogram mechanism [19] can be derived as:

(7) $$K_{P_{1}}=K_{P_{2}}=2\times \frac{12EI}{L^{3}}=\frac{24EI}{L^{3}}\;\;$$

where *E* is Young’s modulus, *I* = *UT*^3^/12 (*U* is the depth and *T* is the thickness) is the moment of inertia of the cross-section area. *L* is the length of identical compliant beams in each compliant parallelogram mechanism.

**Figure 5:**
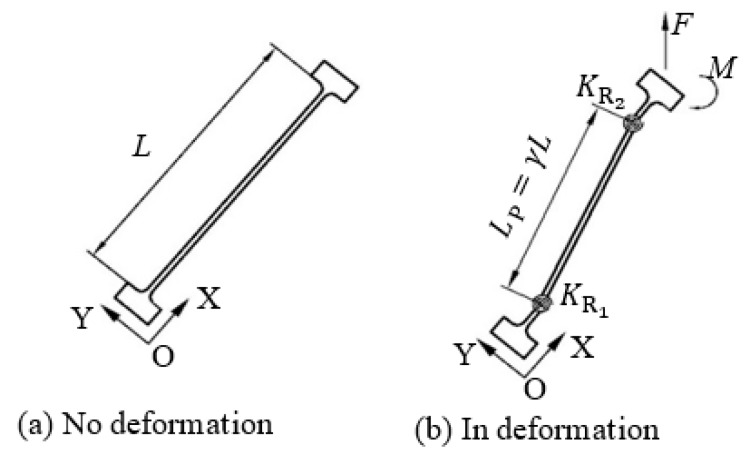
Pseudo-rigid-body model (PRBM) of the inverted compliant beam.

The inverted compliant beam is treated as a fixed-guided beam which has two torsional springs (as R joints) as shown in Figure 5. The stiffness and location of each torsional spring are obtained from the PRBM as reported in [3]:

(8) $$K_{R_{1}}=K_{R_{2}}=2\gamma K_{\theta }\frac{EI}{L}\;$$

where $K_{\theta }$ is the stiffness coefficient. *γ* signifies the characteristic radius factor of the beam curvature. *L* is the length of verted beam, and other symbols are the same as explained above The value of the *K*_θ_ and *γ* are related to the resulting force acting on the end of the fixed-guided beam, which are designated as 0.85 and 2.6, respectively, for a quick esitimation [3].

### 2.4. Kinetostatics

To obtain the relationship between the input force and the input displacement/output displacement (Figure 4), the virtual work principle is performed as below.

The total potential energy generated due to deformation of the compliant beams can be obtained as below:

(9) $$U=2\times \left(\frac{1}{2}K_{P_{2}}y_{\mathrm{in}}{}^{2}\frac{1}{2}K_{R_{2}}\Delta \beta^{2}\frac{1}{2}K_{P_{1}}x_{\mathrm{out}}{}^{2}\frac{1}{2}K_{R_{1}}\Delta \alpha^{2}\right)$$

Based on the principle of virtual work, we have

(10) $$F_{\mathrm{in}}=\frac{dU}{dy_{\mathrm{in}}}-2F_{\mathrm{out}}\frac{x_{\mathrm{out}}}{y_{\mathrm{in}}}\;\;=2K_{P_{2}}y_{\mathrm{in}}+2K_{R_{2}}\frac{\beta_{0}-\arccos\left(\frac{l_{1}+y_{\mathrm{in}}}{L}\right)}{\sqrt{L^{2}-{\left(l_{1}+y_{\mathrm{in}}\right)}^{2}}}+2K_{R_{1}}\frac{\arcsin\frac{l_{1}+y_{\mathrm{in}}}{L}-\alpha_{0}}{\sqrt{L^{2}-{\left(l_{1}+y_{\mathrm{in}}\right)}^{2}}}\;+\left\{2K_{P_{1}}\left[l_{2}-\sqrt{L^{2}-{\left(l_{1}+y_{\mathrm{in}}\right)}^{2}}\right]+F_{\mathrm{out}}\right\}\frac{l_{1}+y_{\mathrm{in}}}{\sqrt{L^{2}-{\left(l_{1}+y_{\mathrm{in}}\right)}^{2}}}$$

where *F*_in_ and *F*_out_ are the forces imposed on the input and output stages, respectively.

By this step, the relationship between the input displacement and the primary output displacement (Equation (4)), and that between the input force and input displacement (Equation (10)) have been established. If the gripper presented in this paper is used for gripping micro-object, the reaction force acting on the jaw is negligible, and thus output force $F_{\mathrm{out}}$ is set to zero in this paper. However, the two parasitic motions of each jaw was not modelled in this paper, which will be analysed by FEA simulations and exprimental testing.

## 3. FEA Simulations and Testing

**Figure 6:**
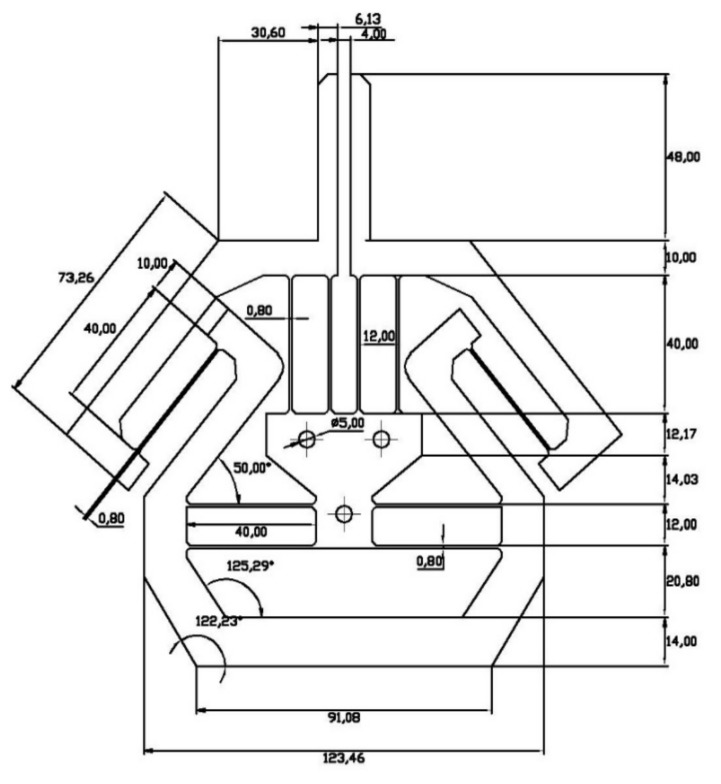
Geometry parameters of the case study (unit in mm).

To verify the analytical model as well as to confirm the performances of the proposed gripper, a specific design as a case study was simulated in Comsol 5.0 (COMSOL Inc., Stockholm, Sweden) and also experimentally tested on the machined prototype. A small fillet of 1.0 mm radius has been introduced at each corner of the beams to alleviate stress concentration. The geometry parameters are provided in Figure 6 with the material of AL 6082 (EU standard) where the modulus of elasticity is *E* = 69 GPa, Poisson’s ratio δ=0.33, and the yield strength [σ]=276 MPa. The thickness (*T*) of the identical compliant beams was determined to be 0.8 mm because of the fabrication limitation. The depth (out-of-plane thickness) of the gripper is 10 mm. The amplification ratio between 1 and 2 is desired in this paper, so the angle of the inverted mechanism was set to be 50°. The jaw length was decided to be 48 mm to facilitate the measurement of the angle of the gripper jaw.

Note that although only the pushing actuation is desired, as explained above, to close the jaws of the compliant gripper (i.e., the closing operation), in this paper we will simulate and experimentally test both the closing and opening operations for comprehensive verifications. We define that a pushing input force is positive (in the Y direction), and that a closing direction of the left jaw is positive (in the X direction).

### 3.1. Simulation

**Figure 7:**
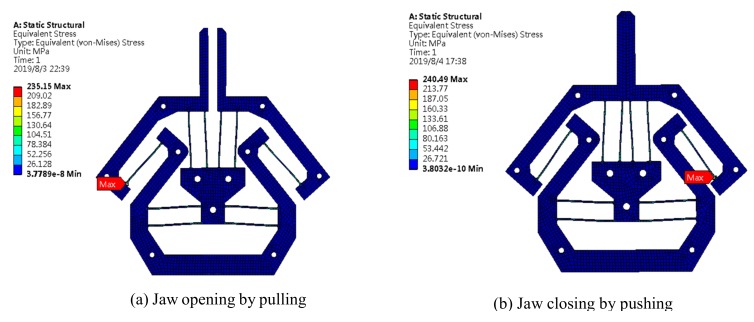
Simulation demonstration.

FEA simulation has been carried out by applying the prescribed input displacement or input force at the bottom of the gripper. The deformed result is as shown in Figure 7. When the gripper is fully closed with a displacement of 2 mm for each jaw (because the distance between two jaws at the home position is 4 mm in this design), the maximal stress is 240.49 MPa which occurs at the end of the inverted beam. With a safety factor of 1.5, the motion range of each jaw can increase ±1.3 mm, so that the gripper can grasp an object with a diameter range between 1.4 mm and 6.6 mm in principle.

Numerical simulation results show good agreement with the analytical results. A detailed comparison among FEA, analytical, and experimental results will be discussed in Section 4.

### 3.2. Experimental Tests

**Figure 8:**
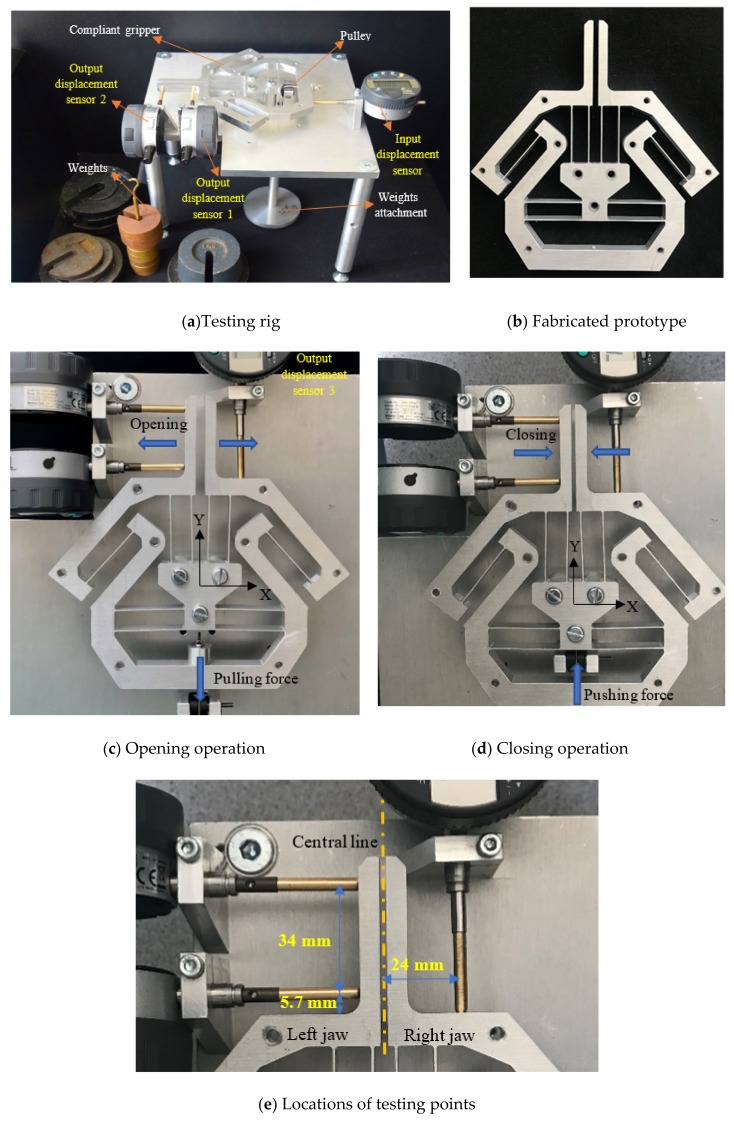
Fabricated prototype and experimental testing rig.

A monolithic gripper was fabricated from a piece of AL 6082 plate through computer numerical control (CNC) milling machining, as shown in Figure 8. AL 6082 was selected because of its lightweight and good mechanical properties. The overall experimental set-up (Figure 8a) is composed of a base, a compliant gripper, an actuation system and displacement gauges. Although the used compliant parallelogram mechanism (jaw) can provide a simple and compact configuration, it makes the jaw have two undesired output motions (i.e., parasitic motions) in addition to its primary output motion. To fully evaluate the output performance of the jaw, the experiment of measuring the primary and parasitic output motions of the jaw was conducted.

Two displacement sensors contacting the left jaw were employed to obtain the (primary) output displacement (in the X direction) and the (parasitic) angle (in the Ө direction) of the jaw. The output displacement in the X direction is calculated using the average of two-point measurements (labeled in Figure 8e). Another displacement sensor at the bottom was used to measure the input displacement (Y direction). Figure 8c demonstrates the set-up of a displacement sensor used to measure the (parasitic) output displacement (in the Y direction) of the right jaw at a specific point (labeled in Figure 8e). All the displacement sensors are digital gauges with a motion resolution of 1 × 10^−3^ mm and a negligible spring force of 0.4–0.7 N, which are produced by Mitutoyo Corporation, Japan. The actuation system is mainly composed of two pulleys and various weights.

Previous FEA results provide a good indication of the maximal input force before the yield occurs. During the testing, a maximum input force of ±100 N was set for the consideration of avoiding the material’s yield failure. Based on the simulation and/or analytical results, an input force of ±100 N can enable a motion range of about ±1.3 mm for each jaw. The achievable maximal stress is highly determined by the material used, and its fabrication quality.

Although the compliant parallelogram mechanism can provide a simple and compact configuration, it still will generate undesired motions (i.e., parasitic motions) in two directions. To further evaluate the output performance of the jaw, the experiment of measuring the parasitic displacement in the Y direction and the parasitic angle in the Ө direction of the jaw was conducted.

## 4. Comparisons

**Figure 9:**
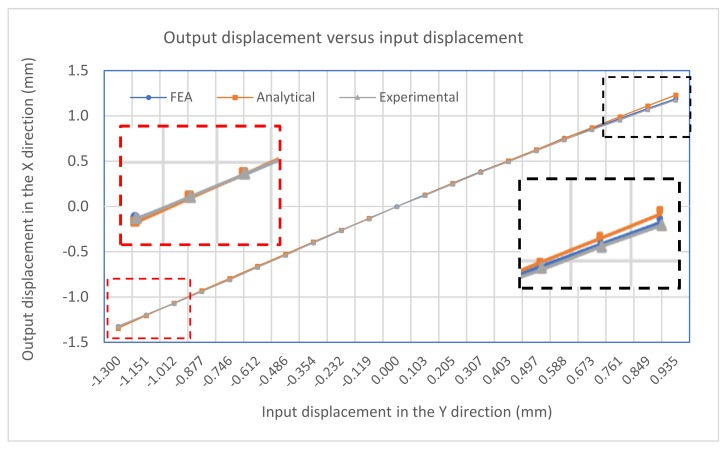
Input-displacement and output-displacement relationship.

**Figure 10:**
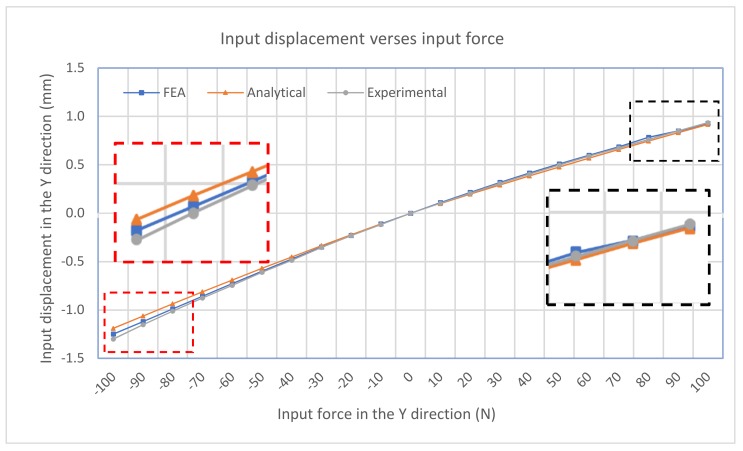
Input-force and output-displacement relationship.

**Figure 11:**
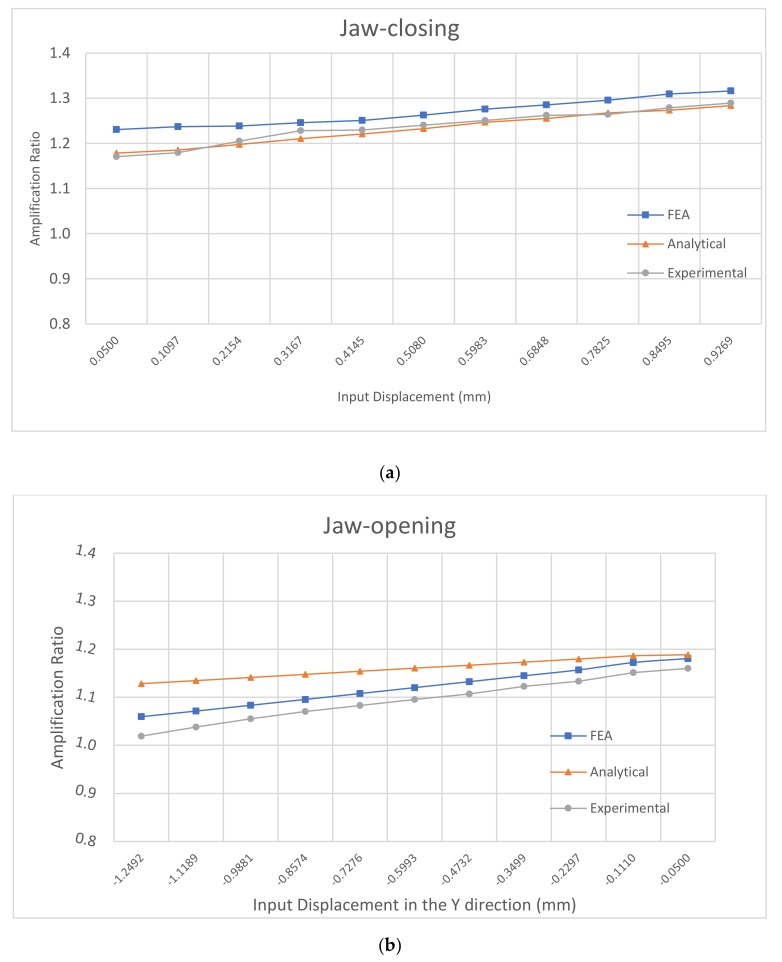
Amplification relation. (**a**) jaw closing; (**b**) jaw opening.

The input-displacement and output-displacement relationship is graphically described in Figure 9 which demonstrates a good agreement between the FEA and analytical results. The maximum difference of the output displacement between the analytical and experimental results is 6.1%. Figure 10 illustrates the relationship between the input-force and input-displacement. There is a small deviation of input displacement between the analytical and experimental results, which is an 8.8% difference. In addtion, the plots also demonstrate the nearly linear relationship between the input force/displacement and the output displacement. Figure 11 summarizes the amplification ratio that varies with the input displacement, as expected in Equation (5). As the input (considering sign) increases, the amplification factor correspondingly increases. The results indicate a good agreement among the three models. The overall amplification ratio ranges from 1 to 1.3. The variable amplification ratio explains why the magnitude of the output displacement in the pushing operation (positive input displacement) is different from that in the pulling operation.

**Figure 12:**
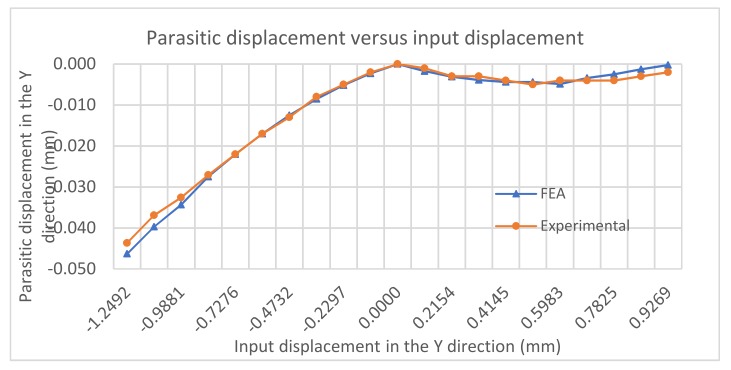
Parasitic motion in the Y direction.

Figure 12 demonstrates the relationship between the input displacement and the parasitic output displacement in the Y direction (at a specific point), which can only be captured by FEA and experiment. We can observe that the FEA results match the experimental ones very well. The parasitic displacement produced in the closing operation is much smaller than that in the opening operation. These findings further confirm that the opening operation should be avoided in this gripper control.

For the opening operation (pulling actuation), the maximum magnitude of parasitic displacement in the Y direction is 0.046 mm, and it falls as the input displacement magnitude decreases. For the closing operation (pushing actuation), the parasitic displacement in the Y direction rises to a peak of 0.0048 mm (10 times smaller than that in the opening operation), before falling steadily. The non-monotonic pattern in the closing operation is due to the increasing tension in the parallelogram mechanism (jaw) as the input displacement increases. The displacement (elastic term) of the parallelogram mechanism caused by the tension (due to pushing actuation) will compensate for its kinematic-term displacement in the Y direction. There are relatively large errors between the experimental results and the FEA results at the ending of the closing operation, since the resolution of the digital gauges does not meet the measurement requirements for very small displacements.

**Figure 13:**
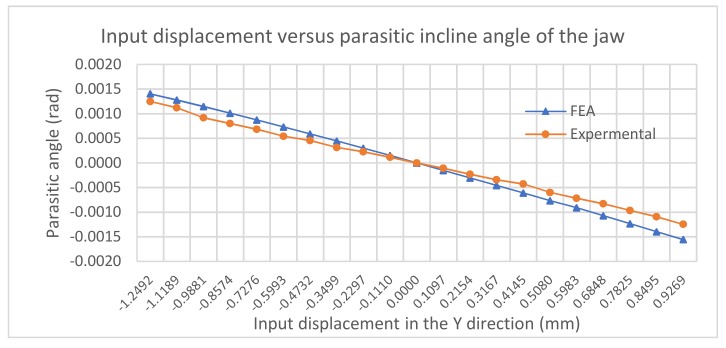
Parasitic rotation in Ө direction.

Figure 13 illustrates the comparison between the FEA and experimental results for the angle of the jaw. As the input displacement magnitude increases, the angle magnitude of the jaw also increases. The results show a good consistency between the FEA and experimental results with the maximal magnitude of less than 0.0015 rad.

**Figure 14:**
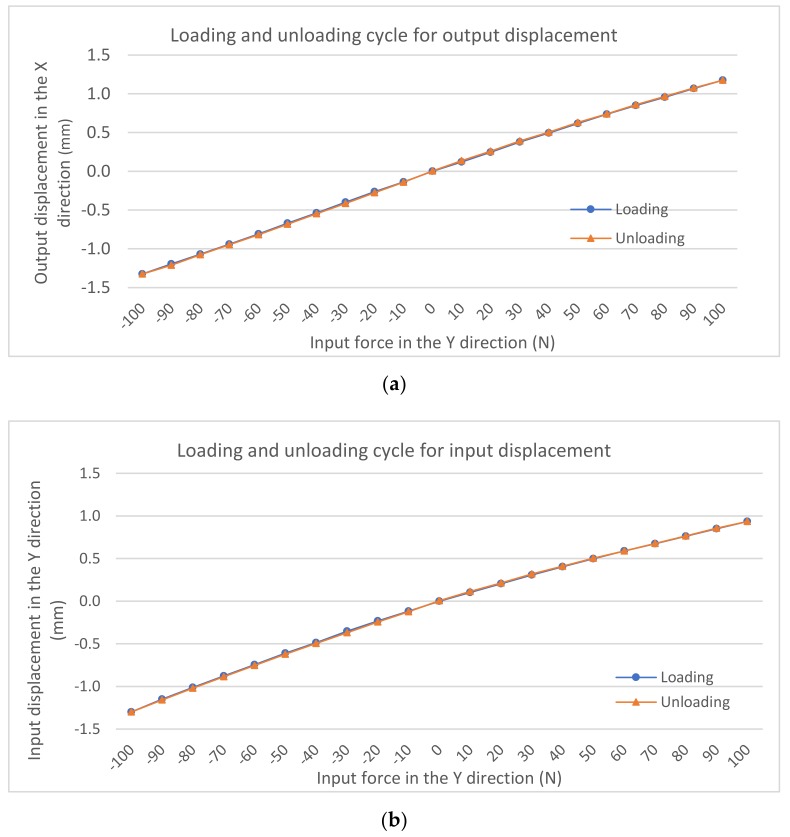
Hysteresis analysis. (**a**) output; (**b**) input.

Hysteresis testing was carried out to further evaluate the characteristics of the gripper under both loading and unloading. The hysteresis characteristics of the output-displacement and input-displacement measurements are graphically presented in Figure 14. The unloading and loading curves coincide well. There is no notable hysteresis effect as seen in Figure 14a,b. For the input displacement measurement, the maximum hysteresis error is 4.5% (9 µm difference) while, for the output displacement measurement, the maximum hysteresis error is 5.6% (13 µm difference).

## 5. Discussions

Through the above comprehensive analysis, we can conclude that the results of FEA, analytical model, and experiment are relatively consistent. The problems associated with parasitic motion of the compliant mechanism, deformation of the assumed rigid bodies, and load-stiffening effect are not considered in the PRBM analytical analysis. The present gripper does not have the minimised parasitic motions of jaws since there was no optimization of the desired motion conducted based on the analytical model. In addition, the manufacturing accuracy and actuation methods are other factors influencing the experimental results.

**Figure 15:**
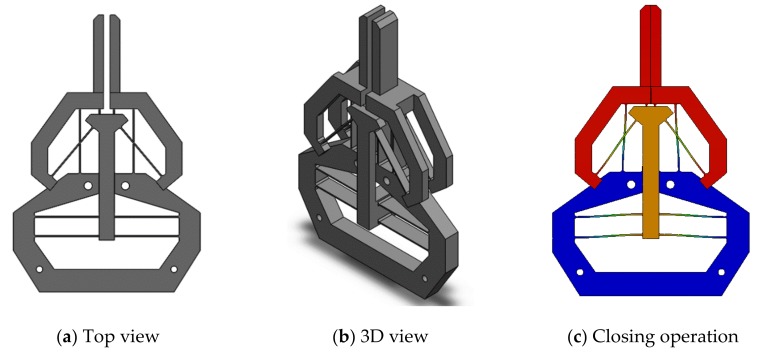
A symmetrical sandwich design for reducing footprint.

In this section, we will discuss two improvements at the cost of losing monolithic configuration: reduction of the footprint and a constant amplifications ratio. In order to further reduce the footprint (increase the compactness) of the proposed compliant gripper, a sandwich design (the first improvement) can be selected to replace the monolithic design, as shown in Figure 15. For half of the gripper, the two parallelogram mechanisms are arranged in the middle layer, while the two identical inverted beams are set up at the top and bottom layers. The improved sandwich design can be fabricated by the assembly or the additive manufacturing.

**Figure 16:**
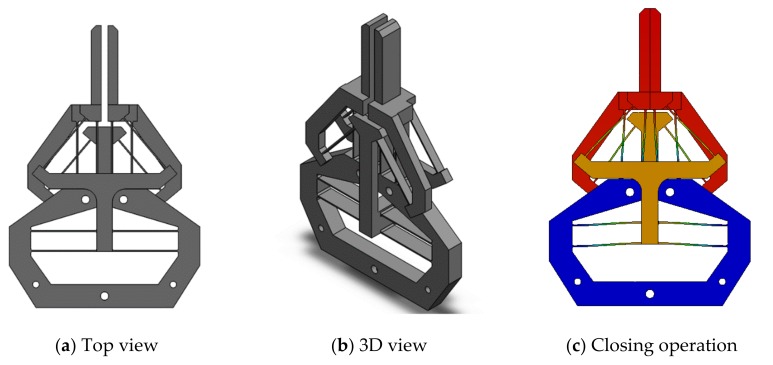
An asymmetrical sandwich design for constant amplification ratio.

**Figure 17:**
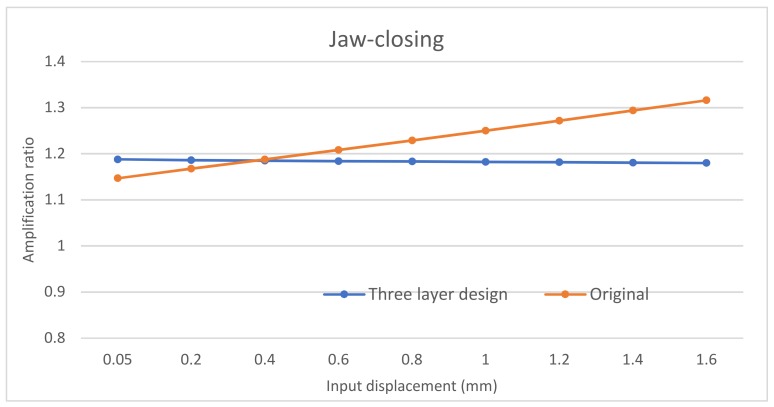
Constant amplification ratio.

The second improvement is to achieve a constant amplification ratio. Compared to the first improved design (Figure 15), the second improved design uses a normally-arranged beam in one side layer and an inverted beam in another side layer, as shown in Figure 16. In this new design, a pushing actuation that leads to an increasing amplification ratio by the inverted beam (Figure 11a) will be compensated via a decreasing amplification ratio by the normally-arranged beam (Figure 11b). The initial FEA comparsion between this improved second design and the original design is shown in Figure 17. We can observe that the new design’s amplification ratio for the jaw-closing case only varies from 1.1546 to 1.1641, which is only 0.8% difference.

## 6. Conclusions

A novel compliant gripper based on a double-slider mechanism has been presented, modelled, and analysed in this paper. It is monolithic, and has a large gripping range without buckling. A combined design method, through combining the PRBM and FEA methods, can be employed to capture accurate performance characteristics of the design before the prototype is made. An alluminium prototype of the gripper was monolithically fabricated by the CNC milling method. Experimental tests were carried out to evaluate the characteristics and effectiveness of the gripper. Several desired performance characteristics of the gripper are summarised as follows:

(1) A large unidirectional grasping range of 1.3 mm for each jaw with a safety factor of 1.5;

(2) A simple structure compared with other existing compliant grippers;

(3) Buckling robustness under large pushing actuation;

(4) An almost linear relationship between input forces/displacement and output displacement.

Future study will focus on nonlinear modeling and optimization of the proposed design, as well as comprehensive analysis of the two improved designs.
